# Long-Term Follow-up of Individual Therapist Delivered and Standardized Hypnotherapy Recordings in Pediatric Irritable Bowel Syndrome or Functional Abdominal Pain

**DOI:** 10.1097/MPG.0000000000003478

**Published:** 2022-05-04

**Authors:** Robyn Rexwinkel, Jeske F.M. Bovendeert, Juliette M.T.M. Rutten, Carla Frankenhuis, Marc A. Benninga, Arine M. Vlieger

**Affiliations:** * From the Emma Children’s Hospital, Amsterdam UMC, University of Amsterdam, Pediatric Gastroenterology, Amsterdam, the Netherlands; the † Department of Pediatrics, St. Antonius Hospital, Nieuwegein, the Netherlands.

**Keywords:** pediatrics, abdominal pain, hypnosis, therapy

## Abstract

We previously showed that standardized home-based gut-directed hypnosis exercises with compact disc (CD) is non-inferior to individual hypnotherapy (iHT) by a therapist in the treatment of children with irritable bowel syndrome (IBS) and functional abdominal pain (syndrome) (FAP(S)). Aim of this follow-up study was to investigate the long-term effects of standardized-hypnosis-recordings at home in comparison to iHT.

**Methods:**

All participants from our previous randomized controlled trial were invited to complete: 1) an online standardized abdominal pain dairy, on which pain frequency and intensity were scored, and 2) an online questionnaire including adequate relief (AR), anxiety/depression scores, somatization, quality of life (QOL), pain beliefs, school and/or work absenteeism and health care utilization.

**Results:**

227 out of 250 (91%) participants completed this study. After a median duration of 6 years follow-up (5.8-6.2), 80.0% in the CD group vs 86.6% in the iHT group reported AR of their abdominal complaints (*P*=.22). Compared to the 1-year follow-up, AR percentages were stable. Treatment success was seen in 67.6% in the CD group vs 71.3% in the iHT group (*P*=.66). Anxiety and depression scores, somatization, pain beliefs, health care utilization and school/work absenteeism also improved significantly in both study groups compared with baseline. No differences were found in QOL-scores.

**Conclusions:**

Both home-based treatment with standardized-hypnosis-recordings and iHT given by a therapist show persisting positive results in more than 80% of children with IBS and FAP(S) after 6 years of follow-up. These results support the rationale for implementation of this easy-to-use, widely available and cost-effective home-treatment in daily practice.

**What is Known**

- Home-based standardized audio hypnosis exercises with compact disc (CD) is non-inferior to individual hypnotherapy (iHT) by a therapist in the treatment of children with irritable bowel syndrome (IBS) and functional abdominal pain (syndrome) (FAP(S)).
- The beneficial effects of iHT are long lasting.
- The long-term effects of standardized hypnosis recordings are unknown.

**What is New**

- This study shows that the beneficial effects of standardized hypnosis recordings are long lasting with 80% of the patients reporting adequate relief of abdominal complaints at 6-year follow-up.
- This supports the implementation of this easy-to-use, widely available, and cost-effective treatment in daily practice.

Irritable bowel syndrome (IBS) and functional abdominal pain (syndrome) (FAP(S)) are highly prevalent pediatric functional gastrointestinal disorders with a worldwide prevalence of 13,5% (1). Both disorders are characterized by chronic or recurrent abdominal pain. In the case of IBS, pain is related to defecation and associated with a change in frequency and/or appearance of stool (2). Quality of life (QOL) is significantly reduced in most children with IBS or FAP(S) (3,4). In addition, these patients have an increased risk for anxiety and/or depression and show higher school and/or work absenteeism rates than their healthy peers (5–7).

One of the most successful therapeutic approaches is gut-directed hypnotherapy (HT) (8). In this treatment, a hypnotic state is induced in which a patient receives suggestions to improve gut function, abdominal pain, general relaxation, ego-strengthening, and coping resources (9–11). Various studies have demonstrated beneficial and long-lasting effects of HT in both adult and pediatric IBS and FAP(S) patients, up to five years after completion of therapy (11–22).

HT has been proven to be effective, but it remains unavailable for many children due to high costs and low availability of well-trained child-hypnotherapists. In addition, visiting a therapist can be time-consuming for both child and parents, causing additional school and work absenteeism. A pilot study in the United States suggested that HT at home using standardized audio exercises could also be an effective treatment option (19). Therefore, a non-inferiority randomized controlled trial (RCT) was conducted in 260 pediatric patients with IBS or FAP(S) to compare the effectiveness of HT with compact disc (CD) recorded self-exercises at home versus individual HT (iHT) given by a qualified therapist. At 1-year follow-up, the home-based HT treatment with CD proved to be non-inferior to iHT by a therapist (17).

Previously we have shown that the beneficial effects of iHT are still present after 5 years follow-up (16). It is unknown if the positive impact of home-based treatment with standardized hypnosis recordings can also persist after such period of time. Therefore, the present study aimed to investigate and compare the long-term effects of audio-self-exercises at home and iHT in children with IBS or FAP(S).

## Methods

### Patients and Procedure

This study describes the long-term follow-up of participants in the Fantasia trial. In this trial, a combination of the Manchester protocol and the protocol of van Tilburg et al was used (9,11,19). In both groups, children were instructed to perform HT exercises during 3 months. In the CD group, children listened to 5 standardized hypnosis recordings, 5 times a week. Children in the iHT group received 6 sessions of 50- to 60 minutes during these 3 months. The same scripts as in the CD group were used; however, adjustments could be made to the individual child. The trial protocol and trial results have been published previously (10,17). All patients participating in the original study who received allocated treatment were eligible for inclusion and were contacted by telephone or email (17). If contact information was outdated, the including medical center and/or the participant’s last known general practitioner were contacted to obtain the most recent contact information. After informed consent was given, participants were asked to complete online questionnaires and an abdominal pain diary. Castor EDC (Castor Electronic Data Capture, Ciwit BV, Amsterdam, the Netherlands) was used to manage the online data.

The requirement for Institutional Review Board approval was waived by the Medical Ethics Committee of the Academic Medical Center on March 26, 2018 (reference number W18_103 # 18.131). Nevertheless, the research met all ethical regulations.

### Outcomes

#### Primary Outcomes

The primary outcomes in this follow-up study were the proportion of participants reporting adequate relief (AR) at long-term follow-up and the proportion of participants in which treatment was successful at long-term follow-up.

For this study, we chose AR as one of our primary outcomes, as recommended by the Rome committee (23). It allows the patient to subjectively combine all relevant symptoms and focus on patients’ own reference system of improvement. This is crucial when evaluating treatment response since this reflects the effect of IBS or FAP(S) symptoms on individual level, independently of symptom severity (24,25). Participants were asked whether they had AR of their IBS- or FAP(S)-related abdominal complaints at long-term follow-up, using a binary outcome (yes/no) (23,24,26).

In order to measure treatment success, patients were asked to keep a 7-day abdominal pain diary in which frequency and intensity of abdominal pain episodes were recorded (11,16,27,28). Pain frequency (PFS) was recorded in minutes of abdominal pain per day (min/day) (scale of 0-21, 0=no pain and 21=pain lasting >120 min/day on 7 consecutive days). Pain intensity (PIS) was scored using an affective facial pain scale (scale of 0-21, 0=no pain at all to 21=indicating severe pain, on 7 consecutive days). Treatment success was defined as ≥50% reduction in both PFS and PIS.

### Secondary Outcomes

Secondary outcomes included depression, anxiety, somatization, health-related QOL, pain beliefs, health care utilization and school and/or work absenteeism in the past four weeks, and the ongoing use of either self-hypnosis or listening to the hypnosis recordings and their reasons for doing so. Details of the instruments used to assess these outcomes are shown online (Supplemental Digital Info http://links.lww.com/MPG/C830).

### Statistical Analysis

For all analyses, an intention-to-treat approach was used. Before the analyses, differences between the group included in the follow-up study and the group lost-to-follow-up were evaluated. A complete case analysis was performed. Numerical data with normal distribution were displayed as mean and standard deviation (SD), and independent two-sample t-tests were used to compare difference between groups. Numerical data with non-normal distribution were displayed as median and interquartile range (IQR), and the Mann-Whitney U tests were used to compare differences between groups. Categorical data were presented as proportions, and comparisons between the two groups were made using the Fisher’s exact test as appropriate. If there were relevant baseline differences between the groups, these variables were included as covariates. Furthermore, a paired sample t-test and a McNemar’s test were conducted to compare changes in baseline and follow‐up in both treatment groups. Logistic regression analyses were performed to evaluate effects of possible covariates for the primary outcomes. Statistical relevance was set at the 0.05 level for all statistical analyses. Statistical analyses were done using R Studio version 3.6.1 (29). The original trial was registered (NTR2725).

## Results

### Participants

Of the 260 participants in the original study, 10 did not receive allocated treatment, resulting in 250 participants being eligible for inclusion in this follow-up study. 227/250 children (91%) agreed to participate. Of the remaining 23 children, 22 children (CD, n = 10; iHT, n = 12) were lost to follow-up and one child refused to participate. Therefore, 115 CD group participants and 112 iHT group participants were included (Supplementary Figure 1 http://links.lww.com/MPG/C830) Both groups had a median follow-up duration of 6 years. There were no differences in demographic, patient and disease characteristics at baseline between the two intervention groups (Table 1). Baseline characteristics of the 23 children not participating in this follow-up study did not differ from those who did (Supplemental Table 1 http://links.lww.com/MPG/C830).

**TABLE 1.** Baseline characteristics of participants by treatment group

	CD group (n = 115)	iHT group (n = 112)
*Demography*		
Female (%)	72	64
IBS/FAP (%)	51/49	48/52
Age at follow-up (years) (*P* < 0.05)	19.4 (2.9)	19.4 (2.8)
Duration of follow-up (years)†	6.0 (5.9 – 6.1)	6.0 (5.8 – 6.2)
*Abdominal pain scores at baseline*		
Pain frequency score*	15.3 (5.4)	14.5 (5.6)
Pain intensity score*	15.2 (4.5)	14.5 (4.5)
Duration of symptoms (years)†	2.4 (1.2 – 5.1)	2.4 (1.1 – 5.2)

CD = compact disc; FAP = functional abdominal pain; IBS = Irritable Bowel Syndrome; iHT = individual hypnotherapy.

* Data are mean (SD).

† Data are median (IQR).

### Primary Outcomes

### Adequate Relief

**Figure 1.**
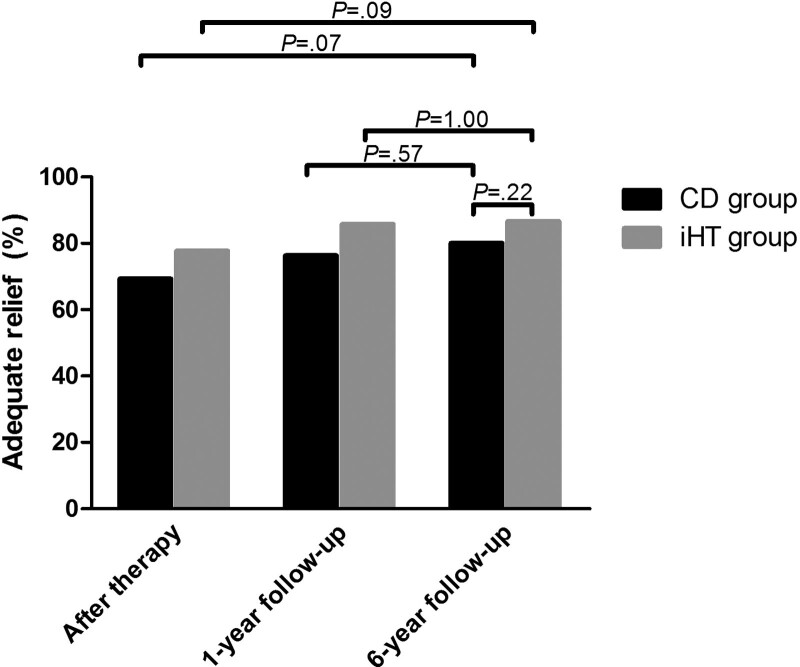
Adequate relief during follow-up. iHT = individual hypnotherapy.

The proportion of children reporting AR at 6-year follow-up was 80.0% in the CD group and 86.6% in the iHT group (*P*=.22; Figure 1). No statistical differences were found in both groups when comparing with AR percentages directly after therapy (CD 69.3% vs 80.0%, *P*=.07; iHT 77.7% vs 86.6%, *P*=.09) and with 1-year follow-up percentages (CD 76.3%, *P*=.57; iHT 85.7%, *P* = 1.00). Type of functional abdominal pain disorder (FAPD) (odds ratio, 1.98; 95%CI, 0.92-4.46), age (odds ratio, 0.82; 95%CI, 0.70- 1.95), gender (odds ratio, 0.43; 95%CI, 0.13-1.17), duration of symptoms at baseline (odds ratio, 0.89; 95%CI, 0.80-9.93), negative beliefs about abdominal pain (odds ratio, 0.60; 95%CI, 0.29-1.20) and anxiety (odds ratio, 0.96; 95%CI, 0.91-1.01) and depression scores (odds ratio, 1.10; 95%CI, 0.92-1.33) did not influence AR at 6-year follow-up in both groups.

### Treatment Success

Fourteen participants did not fill out a complete abdominal pain diary. Therefore, treatment success could only be determined in 213 out of 227 (94%) children (CD, n = 105; iHT, n = 108). At 6-year follow-up, 67.6% of the participants in the CD group were successfully treated, compared to 71.3% participants in the iHT group (*P*=.66). Treatment success at different time points is shown in Table 2.

**TABLE 2.** Treatment success after treatment and at 1- and 6-year follow-up

	CD group* (n = 105)	iHT group* (n = 108)
After therapy	41 (39.0)	54 (50.0)
1-year follow-up	65 (61.9)	76 (70.4)
6-year follow-up	71 (67.6)†	77 (71.3)†

CD = compact disc; iHT = individual hypnotherapy.

* *P* < .001 compared with directly after therapy.

† n (%).

The proportion of children successfully treated during the follow-up period increased significantly in both groups from 39.0% directly after therapy to 67.6% at 6-year follow-up in the CD group (*P*<.001), and from 50.0% to 71.3% in the iHT group (*P*<.001), respectively. No further significant improvement however was seen after 1-year follow-up compared to 6-year follow-up (CD 61.9.%, *P*=.38; iHT 70.4%, *P* = 1.00).

Age (odds ratio, 0.75; 95%CI, 0.65- 0.84) and gender (odds ratio, 0.40; 95%CI, 0.17-0.88) were significantly associated with treatment success at 6-year follow-up in both groups. Girls had 0.4 times less chance than boys to show favorable treatment response. The type of FAPD (odds ratio, 1.40; 95%CI, 0.74-2.69), duration of symptoms at baseline (odds ratio, 1.00; 95%CI, 0.99-1.00), negative beliefs about abdominal pain (odds ratio, 1.00; 95%CI, 0.99-1.00) and anxiety (odds ratio, 0.98; 95%CI, 0.94-1.03) and depression scores (odds ratio, 0.89; 95%CI, 0.78-1.03) did not influence treatment success at 6-year follow-up in both groups.

### Secondary Outcomes

Scores of secondary outcome measures at baseline, directly after therapy and during follow-up are displayed in Supplementary Table 2 http://links.lww.com/MPG/C830. Highlights of these results will be discussed subsequently.

### PFS and PIS

**Figure 2.**
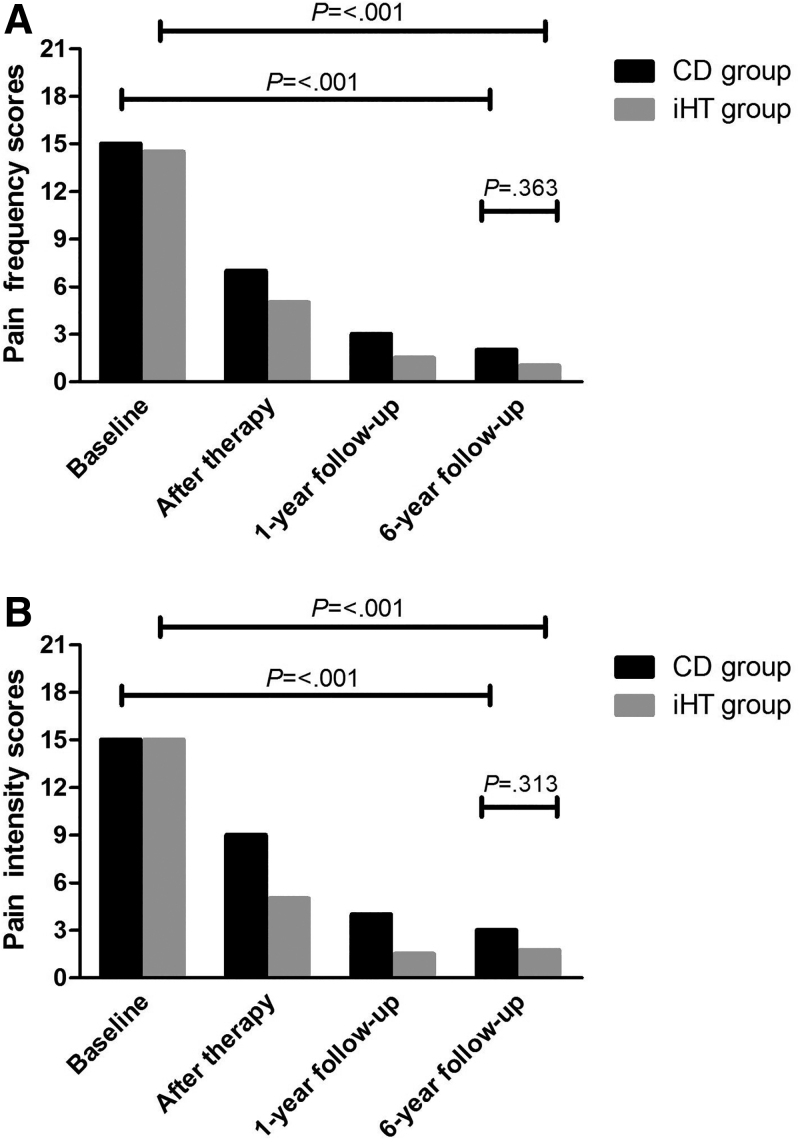
Change in pain frequency (A) and intensity (B) scores during treatment and long-term follow-up. iHT = individual hypnotherapy.

Both PFSs and PISs showed a further decrease compared to 1-year follow-up, although not significant (Figure 2A and 2B). No significant difference was found between the two groups in both median PFSs (CD = 2.0; iHT = 1.0; *U* = 5273, *P*=.363) and PISs (CD = 3.0; iHT = 1.8; *U* = 5227, *P*=.313) at 6-year follow-up.

### Depression and Anxiety

Depression scores at 6-year follow-up did not differ significantly between the CD group (mdn = 2.0) and iHT group (mdn = 3.0) (*U* = 4706.5, *P*=.40). In both intervention groups, depression scores had decreased significantly when compared with baseline (CD: *Z*=-4.325, *P*<.001; iHT: *Z*=-2.582, *P*=.010), but no significant difference when compared to scores at 1-year follow-up (CD: *Z*=-.460, *P*=.645; iHT: *Z*=-.222, *P*=.824).

At 6-year follow-up, anxiety scores were lower in the CD group (mdn = 6.0) compared to the iHT group (mdn = 7.0) (*U* = 4675.0, *P*=.36). Anxiety scores in both study groups were significantly lower compared with baseline (CD: *Z*=-3.441, *P*=.001; iHT: *Z*=-3.427, *P*=.001), but did not differ from the scores at 1-year follow-up (CD: *Z*=-.125, *P*=.901; iHT: *Z*=-.934, *P*=.351).

### Somatization Scores

A significant reduction was found in the total CSI scores, and the separate CSI scores without GI symptoms (non-GI), at 6-year follow-up compared with baseline in the CD group (total: *Z* = .6.161, *P* < .001; non−GI: *Z* = −3.735, *P* = < .001), as well as in the iHT group (total: *Z* = −6.377, *P* < .001; non-GI: *Z* = −3.793, *P* = < .001). Both CSI-scores at 6-year follow-up did not differ from those at 1-year follow-up in the CD- (total: *Z* = −.827, *P* = .408; non-GI: *Z* = −.295, *P* = .768) and iHT group (total: *Z* = −.731, *P* = .465; non-GI: *Z* = -.103, *P* = .918).

Both CSI scores in the iHT group (mdn, total = 11.0; non-GI = 7.0) were lower than those in the CD group (mdn, total = 13.0; non-GI = 8.0) at 6-year follow-up. This difference, however, was not significant (total GI: *U* = 4825, *P*=.585; non-GI: *U* = 4902.5, *P*=.720).

### Health-related QOL

No treatment differences were observed at 6-year follow-up between the CD group and iHT group regarding all ten dimensions of health-related QOL. At 6-year follow-up, the social support and peer scores improved significantly in both groups compared with baseline (P = .002). Also in the iHT group, social acceptance (bullying) scores significantly increased (P = .002). Interestingly, there was a significantly decrease in the dimension self-perception (CD: P = .047; iHT: P = .044). Other long-term follow-up scores did not differ from those at baseline.

### Pain Beliefs

Negative pain belief scores at 6-year follow-up in both treatment groups were significantly lower when compared with baseline (CD: *Z* = −7.456, *P* < 0.001; iHT: *Z* = −8.244, *P* < .001), but did not differ significantly from scores at 1-year follow-up (CD: *Z* = −1.521, *P*<.128; iHT: *Z* = 1.475, *P*<.140). No difference was seen between the two groups.

### Health Care Utilization and Study/work Absenteeism

No differences were found in recent hospital visits or out-of-hospital utilization between the groups, nor were there any differences in absenteeism of study or work (Table 3, Supplemental Digital Content, http://links.lww.com/MPG/C830). 21 Children (CD, n = 11; iHT, n = 10) had missed school/study and/or work in the past month. Of these children, 6 out of 21 (29%) (CD, n = 2; iHT, n = 4) reported no adequate relief and no treatment success.

Six years after the end of therapy, 9.6% of children in the CD group and 29.0% in the iHT group reported to still perform self-HT and/or listen to the audio recordings (*P* < .001). AR and treatment success at 6-year follow-up did not differ from participants in both treatment groups who did or did not practice self-hypnosis (AR, *P* = .93; treatment success, *P* = .78). Reasons for practicing self-hypnosis were stress reduction (n = 16), GI related symptoms (n = 13) or headaches (n = 4).

## DISCUSSION

This study reports the 6-year follow-up of our previously published RCT in which the efficacy of home-based treatment using standardized-hypnosis-recordings was compared to iHT in children with IBS or FAP(S) (17). More than 80% of the participants still reported AR of their abdominal complaints, without significant difference between the two groups. Reductions in total depression and anxiety, somatization and EFCP scores, and improvements in pain beliefs and PFCP scores were also still present in both treatment groups at 6-year follow-up. Health-related QOL-scores were comparable to baseline scores. No difference was found between both study groups regarding school/work absenteeism and health care utilization.

The proportion of participants who had treatment success was 67.6% in the CD group and 71.3% in the iHT group. This ongoing beneficial effect in the iHT group is in accordance with long-term results in children and adults with IBS or FAP(S) (13,16,21). A similar result of treatment success (68%) has been reported previously in a but smaller RCT in children with IBS and FAP(S) at 5-year follow-up, in which a more conservative definition of treatment success (decrease in PFS and PIS >80%) was used (16).

Few studies have examined the effect of hypnotherapeutic self-help approaches for children with IBS or FAP(S). Two pilot studies investigated comparable stand-alone interventions consisting of (1) an instructional video, (2) an audio CD with exercises and, (3) a written instruction for children with IBS or FAP(S). Success rates in these studies varied from 34% directly after treatment to 62% after six months, but to date, no treatment success rates after long-term follow-up like in this study have been reported. It is promising that in our study no difference in efficacy was seen between the two groups, since this home-based hypnosis intervention reduces costs and referral burden compared to iHT provided by a therapist (30). It has been calculated that standardized hypnosis recordings can make hypnosis available in countries with a low number of qualified hypnotherapists. Furthermore, a recent Australian study found that almost all health care professionals believe that no clear evidence for HT exists (31). One reason for this was a lack of access to HT. It seems therefore essential to educate health care professionals on the nature of hypnotherapy and its evidence for efficacy as well as to raise awareness for this easy-access treatment with audio recordings. Currently, online packages with the five HT recordings as used in the original RCT, together with a manual and instruction video, are available in English, Spanish and Dutch (32–34). The fact that, to date, there are not many proven effective therapeutic options for children with IBS or FAP(S) with a lack of evidence for pharmacologic agents as well as for most diets stresses the importance of our findings (35–37). If education on the cause of the abdominal complaints, in combination with possible removal of stress factors and normalization of the diet, does not result in adequate improvement, hypnotherapy, cognitive behavioral therapy or a combination of these two treatments should be proposed by physicians, given that these are the two most effective therapies with long term benefits (8,36,37). However, it is known that parents and children may refuse to engage in psychological services (38). Online HT recordings can be an attractive alternative for them, without the stigma of visiting a psychologist.

In our study, significantly more children in the iHT group reported still using self-hypnosis or listening to the audio recordings. Interestingly, AR and treatment success scores at 6-year follow-up did not differ between those using it and those who did not. This is comparable with another study, where maintained improvement in scores in adults with IBS was not associated with continuing to practice HT (13). It is likely that the therapists had advocated the continuous use of hypnosis exercises for other situations, like stress or other somatic complaints.

Almost all outcomes, like adequate relief, pain beliefs, somatization scores, and feelings of anxiety and depression, had improved in the first year after treatment and had stayed stable since then, emphasizing the long-term effects of hypnotherapy. The quality-of-life scores, however, showed something interesting. Self-perception scores significantly decreased at 6-year follow-up compared with baseline. The only QOL-dimension that significantly improved at 6-year follow-up compared to baseline was social support and peer, while two other QOL-dimensions abated. Possible explanations for this may be the average age at inclusion (13 vs 19) and the high percentage of female participants. There is a decrease in health-related QOL with every age step, which is generally more pronounced in female adolescents than male adolescents (39). Furthermore, the norm data were collected between 2001 and 2004 (40). It might be possible that general QOL among children and adolescents has decreased since then, which is supported by research conducted in the United States where a significant decrease over time was seen in adolescents reporting excellent or very good QOL (41). In addition, the KIDSCREEN-52 has been validated for children aged 8 to 18 years, and it could therefore be hypothesized that the reported QOL-scores do not represent the actual QOL in this older study population (40). However, we chose to use this questionnaire to compare current scores to those from the original study. Final, recent research has shown that a reduction in IBS symptom severity is not paralleled by improvement in QOL in patients with IBS (42).

This study’s strengths are the high number of included patients and the high response rate of 91% at 6-year follow-up. For these reasons, this study comprises one of the most extensive long-term follow-up HT trials in the adult and pediatric FAPD population (11–13,15,16,18–21,28). An explanation for this extremely high response rate may be that online instead of a paper-based versions of the questionnaire and diary were used (43,44). Electronic data collection results in a higher quality of data entry and, consequently, achieve higher adherence rates (44–47). Also, the results have high external validity since the original study recruited children from secondary and tertiary centers in rural and urban areas of the Netherlands. Finally, we chose AR as one of our primary outcomes.

Limitations include the possibility that both IBS’ and FAP(S)’ natural course could have influenced the response in both study arms, since long-term follow-up studies showed that close to 70% of children with IBS or FAP(S) will become free of symptoms over time (48,49). However, most of these studies were performed in primary care and only reported abdominal complaints for ≥3 months as an inclusion criterion, but failed to report the duration of complaints at baseline. Participants in our study already had ≥2 years abdominal complaints and some had been treated previously with standard medical care or psychological therapies for their abdominal pain. In these patients, with long-lasting symptoms, long-term treatment success of standard medical care without HT has been reported to be around 20% (16). Another limitation of this study might be that three patients were diagnosed with an alternative (organic) disorder and four patients underwent other therapies for their abdominal complaints during follow-up. Because of the intention-to-treat principle, these patients were included in analyses, and results on AR and treatment success might be influenced. However, since it only concerns 7/144 (4.9%) participants, we believe this effect is negligible. Furthermore, health care utilization and school/work absenteeism questions only comprised the previous four weeks. This might cause over- or underestimation, depending on the participant, of the actual situation over the past few years. This short period was however, chosen to have an optimal recall period for self-report surveys (50).

## CONCLUSION

In conclusion, this study supports earlier research stating HT to be a highly valuable treatment option with long-lasting beneficial effects in children with IBS or FAP(S). It results in AR in more than 80% of the patients with a considerable decrease in abdominal pain scores and significant improvement in depression, anxiety, somatization, and pain beliefs. This 6-year follow-up provides both patients and health care professionals valuable information about the long-lasting benefits home-based HT by CD exercises and iHT. These results also support the implementation of this easy-to-use, widely available, and cost-effective treatment in daily practice. Future research may be needed to identify what kind of patients will have the highest chance of success with stand-alone hypnosis audio exercises and which patients will benefit more from the personal and individualized interaction with a qualified hypnotherapist.

## Supplementary Material


