# Supplementary material for: Long-Term Follow-up of Individual Therapist Delivered and Standardized Hypnotherapy Recordings in Pediatric Irritable Bowel Syndrome or Functional Abdominal Pain
Source: J Pediatr Gastroenterol Nutr. 2022 May 4;75(1):24–9. doi: 10.1097/MPG.0000000000003478 (PMC9236305; doi:10.1097/MPG.0000000000003478)
Supplement: Supplementary file 1 [file mpg-75-024-s001.pdf]

Details of instruments used to assess secondary outcomes

The short version of the Revised Child Anxiety and Depression Scale (RCADS-25) was used to score anxiety and depression.[24] The RCADS-25 consists of five subscales measuring symptoms of generalized anxiety disorders, separation anxiety disorder, social phobia, panic disorder and major depressive disorder. Each subscale contains five items, scored on a 0 to 3 scale (0=never; 3=always). By summing scores of the four individual anxiety scales, a total score on anxiety was calculated.[10]

The Dutch version of the Children Somatization Inventory (CSI) was used to assess child somatization scores.[25] It contains 35 items, which are scored on a 5-point scale, ranging from not at all (= 0) to a whole lot (= 4). A total score is calculated by summing up the scores of all items with a higher score reflecting a higher intensity in experienced somatic complaints. When leaving out the seven items on gastrointestinal (GI) complaints (bloating, constipation, diarrhea, epigastric and abdominal pain, nausea and vomiting), a separate CSI for non-GI symptoms can be calculated.[10]

To assess health-related QOL, the KIDSCREEN-52 questionnaire was used.[26] The KIDSCREEN-52 contains questions on ten dimensions of health-related QOL. Rasch scores are computed for each dimension and are transformed into T-values; higher scores indicate better health-related QOL and well-being. Since the majority of the participants were >18 years old, some questions were adjusted to be more age-appropriate (i.e. study/work in addition to school).

The Dutch translation of the Pain Beliefs Questionnaire (PBQ) was used to assess negative and positive beliefs about abdominal pain and contains 32 items that need to be rated on a 5-point LikertScale.[27,28] Twenty items concern negative beliefs and can be categorized in five subscales: condition frequency, condition duration, condition seriousness, episode specific

intensity and episode specific duration. Subsequently, the negative beliefs scale is computed by summing the scores of all twenty items. The remaining 12 items assess problem-focused coping potential (PFCP) and emotional-focused coping potential (EFCP). The PFCP and EFCP scale are calculated by averaging the 6 items belonging to each scale. Higher scores on a scale indicate that a participant has those negative beliefs more frequently or has a higher coping potential.

The Dutch Health and Labor Questionnaire (HLQ) was adapted in order to assess the participants' healthcare utilization and school and/or work absenteeism in the past four weeks. Also, children were asked about ongoing use of either self-hypnosis or listening to the hypnosis recordings and their reasons for doing so.

CONSORT

TRANSPARENT REPORTING of TRIALS

CONSORT 2010 Flow Diagram

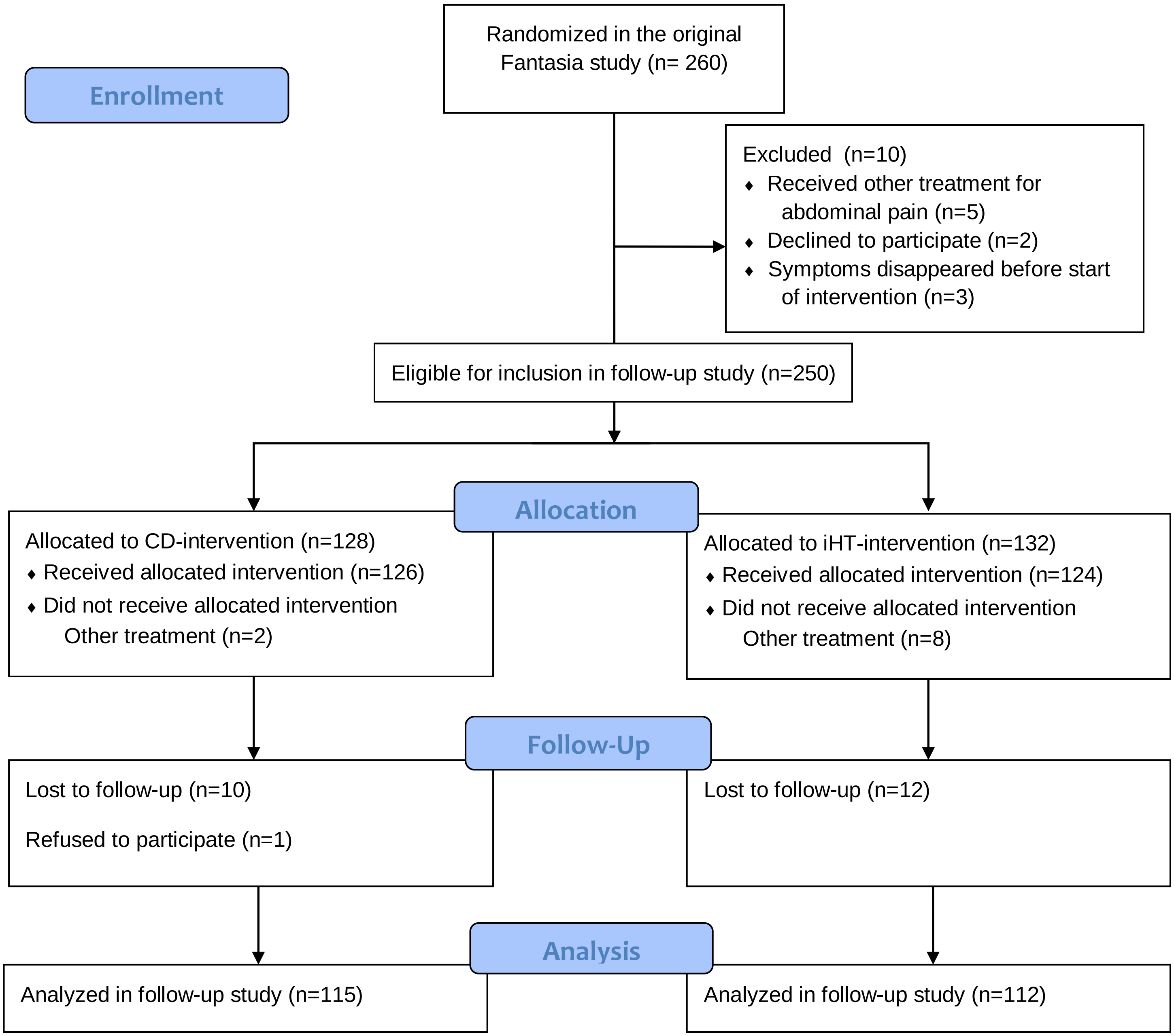

Supplementary Table 1. Baseline characteristics of participants included in the follow-up study versus participants not included in the follow-up study

	**Participants included in follow-up study** (*N*=227)	**Participants not included in follow-up study** (*N*=23)
Demography		
Age (years)[a]	13.4 (2.8)	13.6 (3.0)
Girls (%)	71.8	63.6
Clinical features		
IBS/FAP (%)	51/49	48/51
Duration of symptoms (years)[b]	2.4 (1.2 – 5.1)	2.3 (0.9 – 5.7)
Abdominal pain scores[b]		
Pain Frequency Score	15.0 (11.0 – 21.0)	16.0 (11.0 – 19.0)
Pain Intensity Score	15.0 (12.0 – 18.0)	16.0 (13.0 – 18.0)
Pain beliefs		
Negative pain beliefs[a]	2.2 (0.6)	2.1 (0.7)
Randomisation		
iHT/CD (%)	49/51	60/40

[*] $P<0.05$; [a]Data are mean (SD); [b]Data are median (IQR)
CD = compact disc; iHT = individual hypnotherapy

Supplementary Table 2. Secondary Outcomes During Treatment and Follow-up

	CD group (n=100)				iHT group (n=101)			
	**Baseline**	**After therapy**	**1-year FU**	**6-years FU**	**Baseline**	**After therapy**	**1-year FU**	**6-years FU**
Depression (RCADS-25)[a] (possible score range 0-15)	4.0 (2.0-5.0) (N=100)	3.0 (2.0-5.0) (N=92)	2.0 (1.0-5.0) (N=91)	2.0 (1.0-4.0)* (N=100)	3.0 (2-5) (N=101)	3.0 (1.0-5.0) (N=98)	2.0 (1.0-4.0) (N=96)	3.0 (1.0-4.0)* (N=101)
Anxiety total score (RCADS-25)[a] (possible score range 0-60)	9.5 (5.0-14.0) (N=100)	8.0 (5.5-15) (N=92)	7.0 (2.0-12.0) (N=91)	6.0 (3.0-10.0)* (N=100)	10.0 (5-17) (N=101)	9.0 (4.0-15.0) (N=98)	8.0 (3.0-14.0) (N=96)	7.0 (3.0-12.0)* (N=101)
Somatization: total score (CSI)[a] (possible range 0-140)	22.0 (13.0-33.0) (N=100)	18.0 (9.0-26.5) (N=92)	13.0 (3.5-27.0) (N=91)	13.0 (7.0-20.0)* (N=100)	23.0 (16-33) (N=101)	14.0 (6.0-27.0) (N=98)	11.0 (5.5-23.0) (N=96)	11.0 (6.0-20.0)* (N=101)
Somatization: non-GI symptoms (CSI)[a] (possible score range 0-112)	12.0 (4.0-20.5) (N=100)	8.0 (3.5-17.5) (N=92)	6.0 (1.5-16.0) (N=91)	8.0 (4.0-14.5)* (N=100)	12.0 (7-20) (N=101)	8.0 (3.0-18.0) (N=98)	7.0 (3.0-14.5) (N=96)	7.0 (3.0-14.0)* (N=101)
QoL physical well-being (KIDSCREEN-52)[b] (10th percentile 40.5; 90th percentile 64.6)	45.3 (10.1) (N=100)	48.8 (12.2) (N=92)	46.1 (8.0) (N=91)	45.4 (11.5) (N=100)	44.7 (9.9) (N=100)	47.6 (10.6) (N=92)	46.9 (9.1) (N=90)	43.9 (9.8)† (N=100)
QoL psychosocial well-being (KIDSCREEN-52)[b] (10th percentile 42.6; 90th percentile 68.5)	49.7 (9.5) (N=100)	50.2 (10.2) (N=92)	53.6 (10.2) (N=91)	50.8 (9.5)† (N=100)	48.0 (10.0) (N=100)	51.4 (9.5) (N=92)	53.7 (10.4) (N=90)	49.3 (10.5)† (N=100)
QoL moods&emotions (KIDSCREEN-52)[b] (10th percentile 39.9; 90th percentile 67.5)	50.2 (10.7) (N=100)	50.4 (12.8) (N=92)	50.6 (10.8) (N=91)	51.8 (11.6) (N=100)	47.9 (11.9) (N=100)	52.1 (11.4) (N=92)	52.2 (10.4) (N=90)	49.3 (11.1) (N=100)
QoL self-perception (KIDSCREEN-52)[b] (10th percentile 40.7; 90th percentile 69.8)	52.1 (10.8) (N=100)	52.1 (11.2) (N=92)	52.5 (10.4) (N=91)	49.1 (9.1)* (N=100)	52.2 (10.6) (N=100)	52.8 (10.1) (N=92)	51.2 (11.2) (N=90)	48.9 (9.3)* (N=100)

QoL autonomy (KIDSCREEN-52)[b] (10th percentile 43.9; 90th percentile 68.8)	52.7 (9.8) (N=100)	52.0 (9.1) (N=92)	53.1 (12.0) (N=91)	50.4 (10.0)† (N=100)	51.7 (10.9) (N=100)	53.6 (10.1) (N=92)	53.0 (11.2) (N=90)	51.8 (9.5) (N=100)
QoL relations with parents and home life (KIDSCREEN-52)[b] (10th percentile 41.8; 90th percentile 65.9)	55.7 (9.2) (N=100)	53.7 (9.2) (N=92)	53.6 (10.6) (N=91)	55.1 (9.0) (N=100)	53.2 (10.0) (N=100)	55.2 (9.1) (N=92)	53.5 (9.5) (N=90)	53.4 (10.2) (N=100)
QoL social support&peers (KIDSCREEN-52)[b] (10th percentile 42.3; 90th percentile 66.3)	51.0 (11.7) (N=100)	50.7 (9.5) (N=92)	53.1 (11.0) (N=91)	56.9 (8.6)* (N=100)	49.7 (10.5) (N=100)	51.2 (10.3) (N=92)	54.1 (9.8) (N=90)	55.7 (10.0)* (N=100)
QoL school environment (KIDSCREEN-52)[b] (10th percentile 42.4; 90th percentile 66.1)	54.0 (8.9) (N=100)	53.6 (10.5) (N=92)	53.9 (10.7) (N=91)	53.2 (9.6)† (N=100)	52.2 (9.0) (N=100)	54.3 (12.2) (N=92)	53.6 (10.0) (N=90)	50.6 (9.7)† (N=100)
QoL social acceptance (bullying) (KIDSCREEN-52)[b] (10th percentile 35.2; 90th percentile 58.9)	54.0 (8.2) (N=100)	52.8 (8.2) (N=92)	53.0 (8.7) (N=91)	52.3 (9.9) (N=100)	50.6 (10.6) (N=100)	54.2 (7.7) (N=92)	53.8 (9.1) (N=90)	54.6 (7.8)* (N=100)
QoL financial resources (KIDSCREEN-52)[b] (10th percentile 40.2; 90th percentile 62.9)	56.2 (8.7) (N=100)	56.4 (9.4) (N=92)	55.3 (9.1) (N=91)	55.7 (6.1) (N=100)	55.4 (9.0) (N=100)	57.0 (7.4) (N=92)	56.8 (7.9) (N=90)	54.5 (7.9) (N=100)
Negative pain beliefs (PBQ)[a] (possible score range 0-4)	2.1 (1.8-2.6) (N=98)	1.7 (1.1-2.3) (N=92)	1.3 (0.5-2.1) (N=91)	1.4 (1.2-1.8)* (N=100)	2.3 (2.0-2.8) (N=97)	1.4 (1.1-2.1) (N=96)	1.1 (0.8-1.8) (N=94)	1.4 (1.3-1.5)* (N=100)
Problem focused coping potential (PBQ)[a] (possible score range 0-4)	1.4 (0.7-2.0) (N=98)	2.0 (1.3-2.5) (N=92)	2.3 (1.8-3.3) (N=91)	1.8 (1.5-2.0)*†[#] (N=100)	1.2 (0.8-1.8) (N=97)	2.3 (1.6-3.0) (N=96)	2.3 (2.0-3.2) (N=94)	1.7 (1.3-2.0)*†[#] (N=100)
Emotion focused coping potential (PBQ)[a] (possible score range 0-4)	2.3 (1.7-3.0) (N=98)	2.9 (2.2-3.4) (N=92)	3.3 (3.0-3.8) (N=91)	1.5 (1.3-2.0)*† (N=100)	2.3 (1.7-2.8) (N=97)	2.8 (2.3-3.4) (N=96)	3.3 (2.8-3.7) (N=94)	1.5 (1.3-1.8)*† (N=100)

CD = compact disc; FU = follow-up; iHT= individual hypnotherapy; IQR = interquartile range; QoL = quality of life; SD = standard deviation
[a]= Data are median (IQR); [b]Data are mean (SD); [c]Percentiles based on norm data for children and adolescents aged 8-18 years in the Netherlands
* p<.05 compared to baseline; † p<.05 compared to one year follow-up; [#]significant effect between groups (CD vs iHT)
RCADS-25: lower scores indicate less symptoms of depression/anxiety; CSI: lower scores represent lower intensity of somatic complaints experienced by the child; KIDSCREEN-52: higher scores indicate a better health-related QoL; PBQ: higher scores on a scale indicate that a participant has those negative beliefs more frequently or has a higher coping potential

Supplementary Table 3. Out-of-hospital health care utilization per treatment group (n,%)

	CD group (n=104)	iHT group (n=107)
Health care utilization		
Hospital visits	14 (13.5)	17 (15.9)
General practitioner	25 (24.0)	18 (16.8)
School-doctor	1 (1.0)	2 (1.9)
Psychiatrist	1 (1.0)	4 (3.7)
Phycologist	7 (6.7)	13 (12.1)
Social worker	1 (1.0)	2 (1.9)
Physical therapist	17 (16.3)	17 (15.9)
Hypnotherapist	0 (0)	1 (1.0)
Dietician	1 (1.0)	2 (1.9)
Other*	11 (10.6)	8 (7.5)
	**CD group (n=104)**	**iHT group (n=105)**
Study and/or work absenteeism		
N of participants with absenteeism	11 (11.0)	10 (10.0)
Missed hours per week (median (IQR))	8 (4.0-15.0)	8 (3.5-15.8)
No AR and treatment success	2/11 (18.2)	4/10 (40.0)

*Such as an acupuncturist or naturopath
AR = adequate relief; CD = compact disc; iHT: individual hypnotherapy
$^{*}P<0.05$
